# Assessment of Toxic Effects Associated With Dose-Fractionated Radiotherapy Among Patients With Cancer and Comorbid Collagen Vascular Disease

**DOI:** 10.1001/jamanetworkopen.2020.34074

**Published:** 2021-02-18

**Authors:** Stephanie M. Yoon, Fang-I Chu, Dan Ruan, Michael L. Steinberg, Ann Raldow, Percy Lee

**Affiliations:** 1 Department of Radiation Oncology, University of California, Los Angeles, Los Angeles; 2 Department of Radiation Oncology, MD Anderson Cancer Center, University of Texas, Houston

## Abstract

**Importance:**

The adoption of alternative fractionated radiotherapy regimens for the treatment of patients with cancer and comorbid collagen vascular disease (CVD) is controversial among oncologists because of concerns about potentially severe toxic effects; however, the association between fractionated radiotherapy and toxic effects in the modern era has not been well studied.

**Objective:**

To compare acute and late toxic effects among patients with cancer and comorbid CVD who received dose-fractionated radiotherapy.

**Design, Setting, and Participants:**

This retrospective cohort study examined 197 adult patients with cancer and CVD who received radiotherapy at a single-institution tertiary academic center over a 12-year period (February 1, 2007, to April 30, 2019), with a median follow-up of 23 months (range, 0-108 months). Data were analyzed from February 1 to August 31, 2020.

**Exposures:**

Three dose-fractionated radiotherapy regimens: conventional fractionation (CF; ≤2 Gy per fraction), moderate hypofractionation (MH; >2 Gy to <5 Gy per fraction), and ultrahypofractionation (UH; ≥5 Gy per fraction).

**Main Outcomes and Measures:**

The main outcomes were the incidence and severity of acute and late radiotherapy-associated toxic effects, which were assessed separately by dose-fractionation regimen. Toxic effects occurring within 90 days after radiotherapy completion were considered acute, and toxic effects occurring after that 90-day period were considered late. Secondary goals were to identify covariates associated with toxic effects and to characterize the incidence of CVD symptom flares (defined as worsening clinical symptoms and/or worsening results [transient or permanent] on associated blood tests compared with baseline, as documented by managing physicians) after radiotherapy.

**Results:**

Of 197 patients with cancer and comorbid CVD (mean [SD] age, 69 [12] years; 134 women [68.0%]; and 149 White participants [75.6%]), 80 patients (40.6%) received CF radiotherapy, 55 patients (27.9%) received MH radiotherapy, and 62 patients (31.5%) received UH radiotherapy. The most common CVD diagnoses were rheumatoid arthritis (74 patients [37.6%]), psoriasis (54 patients [27.4%]), systemic lupus erythematosus (34 patients [17.3%]), and scleroderma (8 patients [4.1%]). The most common radiotherapy sites were the breast (48 patients [24.4%]), thorax (25 patients [12.7%]), central nervous system (24 patients [12.2%]), and prostate (23 patients [11.7%]). Data on acute toxic effects were available for 188 patients (95.4%) and missing for 9 patients (4.6%). Data on late toxic effects were available for 142 patients (72.1%) and missing for 55 patients (27.9%). Over 12 years, the unadjusted incidences of severe acute toxic effects associated with CF, MH, and UH radiotherapy were 5.4% (95% CI, 0.3%-10.5%), 7.4% (95% CI, 0.4%-14.4%), and 1.7% (95% CI, 0%-5.0%), respectively. The incidences of severe late toxic effects associated with CF, MH, and UH radiotherapy were 8.3% (95% CI, 1.3%-15.3%), 0%, and 2.2% (95% CI, 0%-6.4%), respectively. No significant associations were found between severe acute or late toxic effects by dose fractionation regimen. In the multivariable analysis, MH radiotherapy was associated with a lower likelihood of developing late toxic effects (odds ratio [OR], 0.21; 95% CI, 0.05-0.83; *P* = .03) compared with CF radiotherapy. Those who received UH radiotherapy had a lower likelihood of experiencing late toxic effects (OR, 0.22; 95% CI, 0.04-1.21; *P* = .08). A total of 19 of 80 patients (23.8%), 15 of 55 patients (27.3%), and 10 of 62 patients (16.1%) experienced CVD symptom flares after receiving CF, MH, and UH radiotherapy, respectively (*P* = .33).

**Conclusions and Relevance:**

In this study, the incidences of unadjusted severe toxic effects over 12 years were less than 10% and were not significantly associated with dose fractionation. When clinically indicated, patients with cancer and comorbid CVD may not require immediate exclusion from the receipt of currently used hypofractionated radiotherapy regimens.

## Key Points

**Question:**

Is dose-fractionation associated with the incidence of toxic effects among patients with cancer and comorbid collagen vascular disease?

**Findings:**

In this cohort study of 197 adults with cancer and comorbid collagen vascular disease, the incidence of severe toxic effects was similar across multiple dose-fractionated radiotherapy regimens, including conventionally fractionated, moderately hypofractionated, and ultrahypofractionated radiotherapy; the unadjusted incidence of severe acute toxic effects was 5%, 7%, and 2%, respectively, while the unadjusted incidence of severe late toxic effects was 8%, 0%, and 2%, respectively. The receipt of hypofractionated radiotherapy was associated with a lower likelihood of developing late toxic effects in the multivariable analysis.

**Meaning:**

This study found that the incidence of severe toxic effects among patients with cancer and comorbid collagen vascular disease was similar across several dose-fractionated radiotherapy regimens.

## Teaser

This cohort study assesses the toxic effects associated with the receipt of dose-fractionated radiotherapy regimens among adult patients with cancer and comorbid collagen vascular disease over a 12-year period.

## Introduction

Collagen vascular disease (CVD) is a heterogeneous group of disorders characterized by the presence of autoantibodies and a dysregulated immune system. Patients with CVD have genetic polymorphisms that, together with environmental triggers, produce widespread inflammation that is associated with injury to connective tissues.^1^ Many patients with CVD have a higher baseline risk of developing site-specific cancers; thus, an increasing proportion of patients is expected to develop cancer as life expectancies increase with the use of new therapies.^2,3,4,5,6,7,8^ At least 50% of all patients with cancer require radiotherapy as part of their care; however, some oncologists have considered a diagnosis of CVD to be a relative contraindication to radiotherapy because of concerns about severe treatment-associated toxic effects.^9,10^

Case studies have historically described disproportionately high incidences of severe toxic effects among patients with CVD who received radiotherapy.^11,12,13,14,15,16^ However, studies using more modern radiotherapy techniques have suggested otherwise.^17,18,19,20,21,22^ The recent CONTRAD (Toxicity After Radiotherapy in Patients With Historically Accepted Contraindications to Treatment) meta-analysis^21^ evaluated 621 patients with cancer, comorbid CVD, and inflammatory bowel disease who received radiotherapy from 1970 to 2018, reporting a 10% to 15% risk of experiencing any grade 3 or higher toxic effects, with a less than 5% risk of experiencing grade 4 toxic effects. The authors concluded that CVD and inflammatory bowel disease were not unconditional contraindications to radiotherapy.

Contemporary radiotherapy clinics are increasingly adopting alternative dose-fractionated radiotherapy into clinical practice for the treatment of many cancers; however, there is controversy about adopting such approaches for patients with cancer and comorbid CVD based on concerns about compounding the risk of toxic effects.^23^ Available studies have reported toxic effects that are primarily associated with conventionally fractionated radiotherapy, and there is minimal data on the association between alternative dose-fractionated regimens and toxic effects in the modern era. Conventional fractionation delivers 1.8 Gy to 2.0 Gy per fraction to the targeted site, which minimizes long-term injury to surrounding healthy tissues while delivering a tumoricidal dose to the target. Hypofractionation is an alternative fractionation regimen that delivers larger (>2 Gy) doses per fraction over fewer treatments. Stereotactic radiosurgery and stereotactic body radiotherapy are highly precise forms of hypofractionation, in which fraction sizes of 5 Gy or higher are delivered conformally to a target site. The advantages of hypofractionation vs conventional fractionation include limiting tumor repopulation, escalating doses for durable local tumor control, requiring a shorter treatment course, and providing cost-effectiveness.^24,25^ However, radiobiological principles state that delivering a larger dose per fraction increases the risk of late toxic effects.^24^

The association between dose fractionation and radiotherapy-associated toxic effects among patients with cancer and comorbid CVD warrants further research. The primary goal of this study was to assess the incidence of acute and late toxic effects separately by dose-fractionated regimen. The secondary goals were to identify covariates associated with toxic effects and to characterize the incidence of CVD symptom flares associated with radiotherapy by dose-fractionated regimen.

## Methods

### Study Population and Design

The electronic database of the University of California, Los Angeles, Department of Radiation Oncology was searched to identify all patients with cancer and concurrent CVD diagnoses using the following keywords: *collagen vascular disease*, *connective tissue disorder*, *rheumatoid arthritis*, *juvenile rheumatoid arthritis*, *systemic lupus erythematosus*, *discoid lupus*, *polymyositis*, *dermatomyositis*, *scleroderma*, *psoriasis*, *psoriatic arthritis*, *ankylosing spondylitis*, and *mixed connective tissue disorders*. Patients were included in the cohort if they initiated radiotherapy in the department. Patients were excluded if they did not receive radiotherapy because this treatment was not clinically indicated, if they were lost to follow-up after consultation, if they chose to receive radiotherapy elsewhere, or if they chose to receive alternative therapies. Radiotherapy treatments were received at a single-institution tertiary academic center from February 1, 2007, to April 30, 2019. The study was approved by the institutional review board of the University of California, Los Angeles. A waiver of informed consent was granted owing to the retrospective nature of the study and the determination that the research involved minimal risk to participants, would not have adverse consequences for the rights and welfare of participants, and could not be practicably conducted without the waiver. This study followed the Strengthening the Reporting of Observational Studies in Epidemiology (STROBE) reporting guidelines for observational cohort studies.

### Data Collection and Review

In the current investigation, data were manually abstracted from electronic medical records by a single radiation oncologist (S.M.Y.) using a uniform data abstraction form to review formatted and narrative data fields. Data were reviewed independently by 3 other radiation oncologists (A.R., P.L., and M.L.S.). Records on age, sex, ethnicity, CVD features, cancer diagnosis, and radiotherapy treatment were obtained. The CVD features included primary diagnosis, activity and symptom flares (defined as worsening clinical symptoms and/or worsening results [transient or permanent] on associated blood tests compared with baseline, as documented by managing physicians) before radiotherapy treatment, and the receipt of steroidal medications or disease-modifying antirheumatic drugs. Data on cancer diagnosis included histologic characteristics, cancer stage, year of diagnosis, and disease status (primary or recurrent). Radiotherapy treatment details included technique, receipt of previous treatments, dose-fractionated regimen, treatment dates, planning target volume, concurrent use of systemic therapies, date of last follow-up, and survival status. The biological effective dose was calculated to compare the total dose delivered across different fractionation approaches with a common numeric score (eMethods in the Supplement).

Dose-fractionated radiotherapy regimens were defined as conventional fractionation (CF; ≤2 Gy per fraction), moderate hypofractionation (MH; >2 to <5 Gy per fraction), and ultrahypofractionation (UH; ≥5 Gy per fraction). Toxic effects occurring within 90 days after treatment completion were considered acute, and any toxic effects occurring after that 90-day period were considered late. Radiotherapy-associated toxic effects were graded using the *Common Terminology Criteria for Adverse Events*, version 5^26^ (grade range, 1-5, with 1 indicating a mild or asymptomatic event and 5 indicating death). Toxic effects with grades of 3 or higher were considered severe.

### Statistical Analysis

Descriptive statistics were reported for demographic characteristics, clinical features, and toxic effects incidence by dose fractionation group. We obtained 95% CIs for proportion for the primary end point via a simple asymptotic formula based on normal approximation. To assess the association between dose fractionation groups and categorical covariates, χ^2^ or Fisher exact tests were used, as appropriate. To assess the association between dose fractionation groups and continuous covariates, 1-way analysis of variance was used. Kruskal-Wallis test was used to assess the difference in median of continuous variable among groups.

Univariate logistic regression analysis was performed to evaluate the association between acute and late toxic effects and the dose-fractionated regimen. Multivariable logistic regression analysis was conducted to assess this association, adjusting for other covariates including age, race, primary CVD diagnosis, CVD activity, cancer diagnosis, radiotherapy treatment site, use of concurrent systemic therapies, history of radiotherapy, planning target volume, and year of radiotherapy treatment. The overall significance of a categorical variable in the multivariable logistic regression model was assessed through the likelihood ratio test.

The cumulative incidence of CVD symptom flares by dose fractionation group was estimated, with death as the competing event. The association between CVD symptom flares and dose fractionation group was assessed using Fine-Gray competing risk regression models, with and without adjusting for potential confounding variables, CVD activity, and medication receipt. The significance threshold was set at *P* = .05, and all tests were 2-sided. All analyses were conducted using R software, version 3.6.0 (R Foundation for Statistical Computing), with the cmprsk package.^27,28^ Data were analyzed from February 1 to August 31, 2020.

## Results

### Patient Characteristics

The initial departmental database query identified 474 patients with cancer and a CVD diagnosis. A total of 197 patients (mean [SD] age, 69 [12] years; 134 women [68.0%]; and 149 White participants [75.6%]) were included in the study cohort, representing 247 radiotherapy treatment courses; 277 patients were excluded from the cohort because radiotherapy was not clinically indicated (170 patients [61.4%]), radiotherapy was received at a location other than our facility (11 patients [4.0%]), an alternative cancer treatment was chosen (1 patient [0.4%]), or loss to follow-up occurred after consultation (89 patients [32.1%]). Only 6 excluded patients (2.2%) received a recommendation against receiving radiotherapy because of CVD activity.

Among 197 patients, the most common CVDs were rheumatoid arthritis (74 patients [37.6%]), psoriasis (54 patients [27.4%]), and systemic lupus erythematosus (34 patients [17.3%]). Eight patients (4.1%) had scleroderma. Eighty patients (40.6%) received CF radiotherapy, 55 patients (27.9%) received MH radiotherapy, and 62 patients (31.5%) received UH radiotherapy. The most common radiotherapy sites were the breast (48 patients [24.4%]), thorax (25 patients [12.7%]), central nervous system (24 patients [12.2%]), and prostate (23 patients [11.7%]). Characteristics of patients by dose fractionation group are summarized in Table 1. Differences in age were found among dose fractionation groups (mean [SD] age, 67 [12] years in the CF group vs 67 [14] years in the MH group vs 72 [10] years in the UH group). While most of the patients in all groups were White and female, the UH group included a numerically lower proportion of female participants (32 women [51.6%] vs 56 women [70.0%] in the CF group and 46 women [83.6%] in the MH group). Although no significant differences were observed in CVD subtypes or activity between dose fractionation groups, a lower proportion of participants in the UH group (29 patients [46.8%]) were receiving any CVD medications compared with those in the CF group (56 patients [70.0%]) and the MH group (36 patients [65.5%]).

**Table 1.** Baseline Patient Characteristics by Dose-Fractionated Radiotherapy Regimen

Characteristic	No. (%)			*P* value^a^
	Conventional fractionation (n = 80)	Moderate hypofractionation (n = 55)	Ultrahypofractionation (n = 62)	
Age, mean (SD), y	67 (12)	67 (14)	72 (10)	.02
Female sex	56 (70.0)	46 (83.6)	32 (51.6)	<.001
White race	59 (73.8)	44 (80.0)	46 (74.2)	.67
CVD diagnosis				
Rheumatoid arthritis	25 (31.3)	21 (38.2)	28 (45.2)	.12
Systemic lupus erythematosus	20 (25.0)	6 (10.9)	8 (12.9)	
Psoriasis	26 (32.5)	15 (27.3)	13 (21.0)	
Scleroderma	1 (1.2)	4 (7.3)	3 (4.8)	
Other	8 (10.0)	9 (16.4)	10 (16.1)	
CVD medication received				
Any^b^	56 (70.0)	36 (65.5)	29 (46.8)	.01
Steroid	15 (18.8)	7 (12.7)	9 (14.5)	.61
CVD activity during radiotherapy				
Controlled	60 (75.0)	41 (74.5)	47 (75.8)	.75
Low	19 (23.8)	12 (21.8)	12 (19.4)	
Moderate to high	1 (1.2)	2 (3.6)	3 (4.8)	
Cancer diagnosis^c^				
Breast	22 (27.5)	29 (52.7)	4 (6.5)	<.001
Lung	4 (5.0)	3 (5.5)	14 (22.6)	
Genitourinary	4 (5.0)	1 (1.8)	21 (33.9)	
Head and neck	13 (16.3)	5 (9.1)	4 (6.5)	
Radiotherapy site^c^				
Breast	22 (27.5)	25 (45.5)	1 (1.6)	<.001
Thorax	8 (10.0)	3 (5.5)	14 (22.6)	
Prostate	4 (5.0)	0	19 (30.6)	
Central nervous system	10 (12.5)	5 (9.1)	9 (14.5)	
Treatment technique				
3D-CRT^d^	27 (33.8)	32 (58.2)	0	<.001
IMRT	47 (58.8)	12 (21.8)	0	
Electron therapy	1 (1.3)	2 (3.6)	0	
Brachytherapy	0	8 (14.5)	14 (22.6)	
SBRT	0	0	48 (77.4)	
PTV, mean (SD), mL	3648 (17 190)	668 (1665)	156 (492)	.18
BED-10, mean (SD), Gy	64.2 (16.7)	54.6 (15.5)	84.8 (11.2)	.03

Abbreviations: 3D-CRT, 3-dimensional conformal radiotherapy; BED-10, biological effective dose with α/β ratio of 10; CVD, collagen vascular disease; IMRT, intensity-modulated radiotherapy; PTV, planning target volume; SBRT, stereotactic body radiotherapy.

^a^ For categorical variables, *P* values were obtained using χ^2^ or Fisher exact tests, as appropriate. For continuous variables, *P* values were obtained using 1-way analysis of variance.

^b^ Any CVD medication includes both disease-modifying antirheumatic drugs and steroidal medications. Among 80 patients receiving conventionally fractioned radiotherapy, 55 patients receiving moderately hypofractionated radiotherapy, and 62 patients receiving ultrahypofractionated radiotherapy, 24 patients, 19 patients, and 33 patients, respectively, were not receiving any medications to specifically treat CVD.

^c^ The 4 most common sites in the cohort are listed.

^d^ Data were missing for 5 patients receiving conventionally fractionated radiotherapy and 1 patient receiving moderately hypofractionated radiotherapy.

Substantial differences in cancer diagnoses, radiotherapy sites, and treatment techniques were observed among the 3 groups. Participants with breast cancer received CF radiotherapy (22 patients [27.5%]) or MH radiotherapy (29 patients [52.7%]) compared with UH radiotherapy (4 patients [6.5%]), while more patients with lung and genitourinary cancers (specifically prostate cancer) received UH radiotherapy (14 patients [22.6%] and 21 patients [33.9%], respectively) compared with CF radiotherapy (4 patients [5.0%] and 4 patients [5.0%], respectively) and MH radiotherapy (3 patients [5.5%] and 1 patient [1.8%], respectively), likely reflecting extant departmental practice patterns. Three-dimensional conformal radiotherapy and intensity-modulated radiotherapy treatment techniques were primarily used for CF radiotherapy (27 patients [33.8%] and 47 patients [58.8%], respectively) and MH radiotherapy (32 patients [58.2%] and 12 patients [21.8%], respectively), while brachytherapy (14 patients [22.6%]) and stereotactic body radiotherapy (48 patients [77.4%]) were used for UH radiotherapy. The mean planning target volume was smaller among both of the hypofractionation groups (mean [SD], 668 [1665] mL for the MH group and 156 [492] mL for the UH group) compared with the CF group (mean [SD], 3648 [17 190] mL) but was not significantly different (*P* = .18) among the 3 groups. The biological effective dose was significantly different among dose fractionation groups (mean [SD], 64.2 [16.7] Gy for the CF group; 54.6 [15.5] Gy for the MH group; and 84.8 [11.2] for the UH group; *P* = .03).

### Outcomes

**Figure 1.**
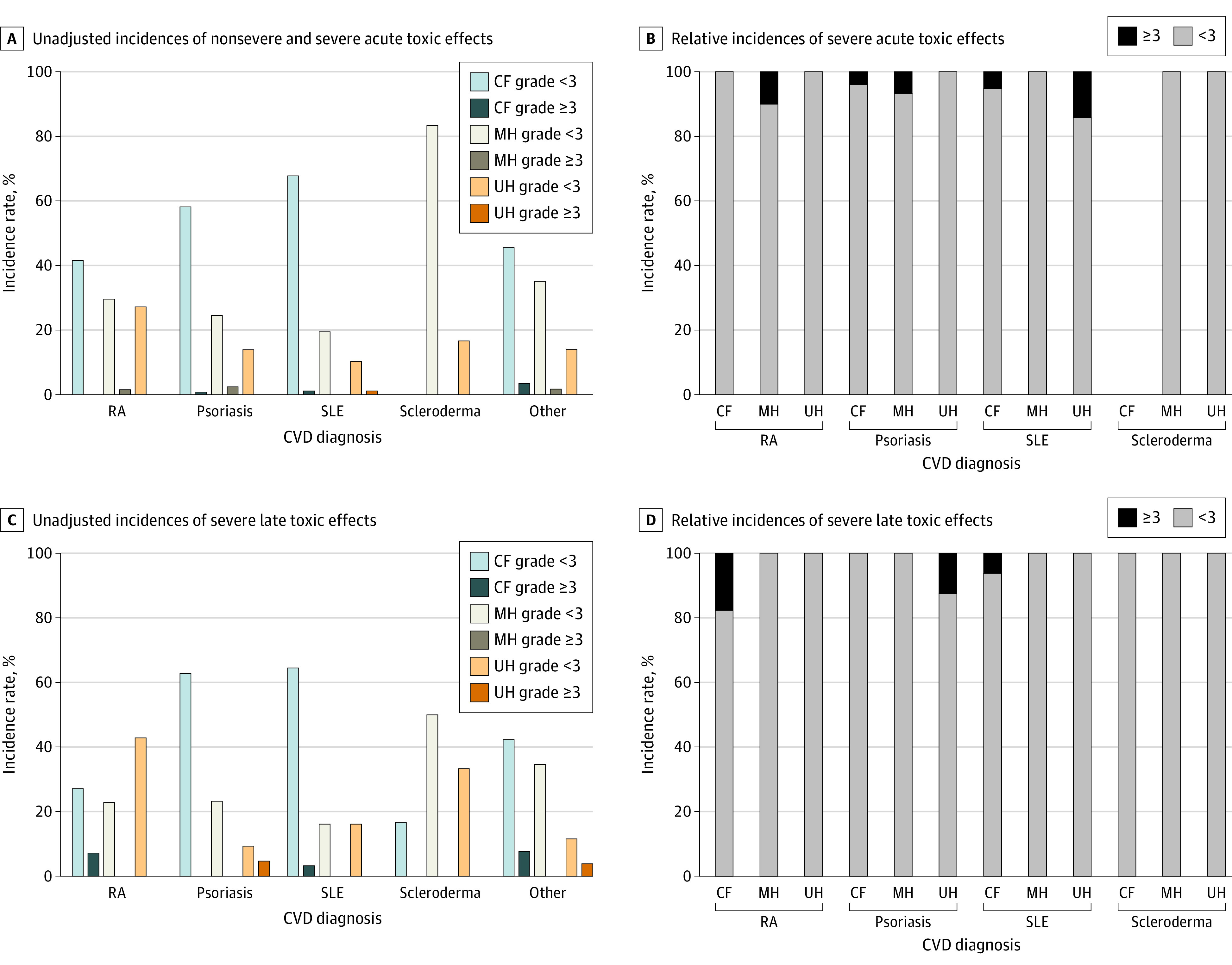
Toxic Effects by Dose-Fractionated Radiotherapy Regimen and CVD Diagnosis A, Unadjusted incidence of nonsevere and severe acute toxic effects. Nonsevere toxic effects were those lower than grade 3, and severe toxic effects were those at grade 3 or higher. B, Relative incidence of severe toxic effects among patients who developed an acute adverse reaction. C, Unadjusted incidence of severe late toxic effects. D, Relative incidence of severe late toxic effects. CF indicates conventional fractionation; CVD, collagen vascular disease; MH, moderate hypofractionation; RA, rheumatoid arthritis; SLE, systemic lupus erythematosus; and UH, ultrahypofractionation.

The median time from the start of radiotherapy to the date of the last follow-up was 23 months (range, 0-108 months). The median follow-up times after receipt of CF, MH, and UH radiotherapies were 24, 24, and 16 months, respectively (*P* = .20). Of 197 patients, 1 patient (0.5%) did not complete treatment, and 4 patients (2.0%) had incomplete records from which treatment completion could not be determined. Data on acute toxic effects were available for 188 patients (95.4%) and missing for 9 patients (4.6%); data on late toxic effects were available for 142 patients (72.1%) and missing for 55 patients (27.9%). Table 2 details the unadjusted incidence of acute and late toxic effects by dose fractionation group. Among participants with available data on acute toxic effects, 9 patients (4.8%) experienced severe toxic effects. The unadjusted incidences of severe acute toxic effects associated with CF, MH, and UH radiotherapies were 5.4% (95% CI, 0.3%-10.5%), 7.4% (95% CI, 0.4%-14.4%), and 1.7% (95% CI, 0%-5.0%), respectively. Severe acute toxic effects were not significantly associated with dose fractionation regimen or treatment technique. The incidences of unadjusted and relative severe acute toxic effects by dose fractionation group across CVD diagnoses are shown in Figure 1A, Figure 1B, and Figure 1C. Of 397 identified acute toxic effects, the most common were dermatitis (81 patients [20.4%]), pain (56 patients [14.1%]), and fatigue (52 patients [13.1%]).

**Table 2.** Unadjusted Incidence of Acute and Late Toxic Effects by Grade Over 12 Years, Stratified by Dose-Fractionated Radiotherapy Regimen

Toxic effect	No. (%)		
	Conventional fractionation	Moderate hypofractionation	Ultrahypofractionation
**Acute**			
Patients with missing toxic effects data, No.	6	1	2
Patients with available toxic effects data			
Total patients, No.	74	54	60
Grade^a^			
0	7 (9.5)	5 (9.3)	26 (43.3)
1	36 (48.6)	28 (51.8)	22 (36.7)
2	27 (36.5)	17 (31.5)	11 (18.3)
3	4 (5.4)	4 (7.4)	1 (1.7)
4	0	0	0
5	0	0	0
**Late**			
Patients with missing toxic effects data, No.	20	19	16
Patients with available toxic effects data			
Total patients, No.	60	36	46
Grade^a^			
0	14 (23.3)	17 (47.2)	24 (52.2)
1	30 (50.0)	13 (36.1)	13 (28.3)
2	11 (18.3)	6 (16.7)	8 (17.4)
3	4 (6.7)	0	1 (2.2)
4	1 (1.7)	0	0
5	0	0	0

^a^ Toxic effects were graded using the *Common Terminology Criteria for Adverse Events*, version 5^26^ (grade range, 1-5, with 1 indicating a mild or asymptomatic event and 5 indicating death). Toxic effects with grades of 3 or higher were considered severe.

The unadjusted incidences of severe late toxic effects associated with CF, MH, or UH radiotherapies were 8.3% (95% CI, 1.3%-15.3%), 0%, and 2.2% (95% CI, 0%-6.4%), respectively. Severe late toxic effects were not significantly associated with dose fractionation or treatment technique. One of 8 patients (12.5%) with psoriasis and late toxic effects developed severe toxic effects after receiving UH radiotherapy, while no patients developed severe toxic effects after receiving CF or MH radiotherapy (Figure 1D). No patients with rheumatoid arthritis, systemic lupus erythematosus, or scleroderma developed severe toxic effects after receiving MH or UH radiotherapy. Three of 17 patients (17.6%) with rheumatoid arthritis developed severe toxic effects after receiving CF radiotherapy. Of 176 identified late toxic effects, the most common were pain (18 patients [10.2%]), skin hyperpigmentation (11 patients [6.3%]), and superficial soft tissue fibrosis (8 patients [4.6%]).

**Figure 2.**
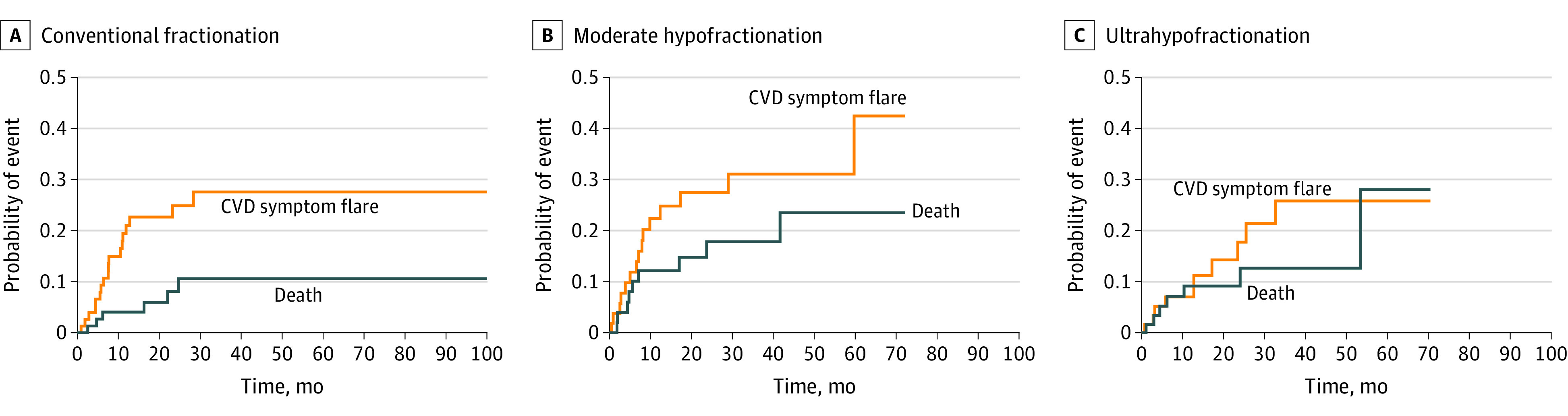
Cumulative Incidence of CVD Symptom Flare by Dose-Fractionated Radiotherapy Regimen Death was used as the competing event. CVD indicates collagen vascular disease.

Nineteen of 80 patients (23.8%; 95% CI, 14.5%-33.1%) receiving CF radiotherapy, 15 of 55 patients (27.3%; 95% CI, 15.5%-39.1%) receiving MH radiotherapy, and 10 of 62 patients (16.1%; 95% CI, 7.0%-25.2%) receiving UH radiotherapy experienced a CVD symptom flare. Dose fractionation regimen and treatment technique were not significantly associated with CVD symptom flares. The cumulative incidences of CVD symptom flare by dose fractionation group are shown in Figure 2. Overall, 22 of 197 patients (11.2%) died without experiencing a CVD symptom flare. No significant association was found between CVD symptom flare and dose fractionation regimen in both the unadjusted and adjusted Fine-Gray models. In the adjusted model, CVD activity was associated with a greater risk of experiencing a CVD symptom flare, with a subdistribution hazard ratio of 2.10 (95% CI, 1.11-3.99; *P* = .02). Of 44 participants who experienced a CVD symptom flare, 23 patients (52.3%) adjusted or changed their CVD medications (either disease-modifying antirheumatic drugs or steroidal medications). Five of 44 patients (11.4%) were diagnosed with CVD after completing radiotherapy.

### Logistic Analyses

In the univariate analysis, UH radiotherapy was significantly associated with a lower likelihood of acute toxic effects (odds ratio [OR], 0.14; 95% CI, 0.05-0.35; P < .001) compared with CF radiotherapy. Both MH and UH regimens were associated with a lower likelihood of late toxic effects compared with CF radiotherapy (for MH radiotherapy, OR, 0.34 [95% CI, 0.14-0.83]; *P* = .02; for UH radiotherapy, OR, 0.28 [95% CI, 0.12-0.64]; *P* = .003).

In the multivariable analysis, dose fractionation was associated with late toxic effects by the likelihood ratio test. The OR was 0.21 (95% CI, 0.05-0.83; *P* = .03) for MH radiotherapy and 0.22 (95% CI, 0.04-1.21; *P* = .08) for UH radiotherapy compared with CF radiotherapy. The treatment site was also associated with late toxic effects by the likelihood ratio test (*P* = .006). Radiotherapy that targeted musculoskeletal sites (of which 16 of 22 treatments were directed toward nonspinal bone) had an OR of 0.02 (95% CI, 0-0.36; *P* = .008) compared with radiotherapy that targeted the breast. Patients receiving multiple courses of radiotherapy had an OR of 8.58 (95% CI, 1.33-55.52; *P* = .02) for late toxic effects (Table 3).

**Table 3.** Multivariable Analysis of Factors Associated With Acute and Late Toxic Effects

Variable	Acute toxic effects^a^		Late toxic effects	
	OR (95% CI)	*P* value	OR (95% CI)	*P* value
Intercept	1215.15 (8.09-182 613.42)	.005	4.27 (0.07-258.65)	.49
Age	0.98 (0.93-1.04)	.55	0.98 (0.94-1.02)	.30
Race	0.40 (0.11-1.56)	.19	0.65 (0.20-2.09)	.47
Primary CVD diagnosis^b^				
Psoriasis	2.23 (0.46-10.74)	.32	1.03 (0.26-4.11)	.97
Rheumatoid arthritis	1.12 (0.27-4.72)	.88	2.56 (0.70-9.29)	.15
Scleroderma	0.56 (0.07-4.25)	.58	0.31 (0.03-3.18)	.33
Systemic lupus erythematosus	2.23 (0.38-13.26)	.38	1.31 (0.28-6.20)	.73
CVD activity	1.03 (0.31-3.43)	.97	0.76 (0.22-2.68)	.68
Cancer diagnosis^c^				
Central nervous system	0.28 (0.02-3.17)	.30	1.41 (0.11-18.78)	.79
Gastrointestinal	1.79 (0.05-66.35)	.75	0.65 (0.03-12.23)	.77
Genitourinary	0.19 (0.01-2.41)	.20	0.64 (0.05-8.82)	.74
Gynecological	0.02 (0-0.40)	.01	0.40 (0.03-5.93)	.51
Head and neck	1.58 (0.14-17.70)	.71	2.83 (0.10-79.34)	.54
Lung	0.59 (0.07-5.30)	.64	6.05 (0.54-67.55)	.14
Hematologic	2.00 (0.09-43.49)	.66	0.05 (0-2.22)	.12
Sarcoma	4.61 (0.13-159.00)	.40	5.10 (0.24-109.51)	.30
Skin	0.08 (0-1.43)	.08	1.08 (0.05-25.43)	.96
Thoracic (other)	4.79 (0.16-140.14)	.36	3.69 (0.13-104.84)	.44
Radiotherapy site^c^				
Central nervous system	0.29 (0.03-2.90)	.30	0.10 (0.01-1.08)	.06
Gastrointestinal	1.19 (0.02-87.15)	.94	0.88 (0.04-18.89)	.94
Gynecological	10.15 (0.41-250.34)	.16	2.75 (0.16-46.74)	.48
Head and neck	0.28 (0.03-3.01)	.30	17.96 (0.36-893.95)	.15
Musculoskeletal	0.03 (0-0.44)	.009	0.02 (0-0.36)	.008
Prostate	0.57 (0.04-8.23)	.68	1.75 (0.12-24.66)	.68
Skin	1.23 (0.06-23.61)	.89	1.06 (0.04-25.22)	.97
Thorax	0.15 (0.01-1.60)	.12	0.29 3 (0.03-2.82)	.29
Concurrent systemic treatment^d^				
Chemotherapy	3.21 (0.21-48.37)	.40	1.20 (0.13-1.52)	.87
Hormone therapy	1.86 (0.15-22.42)	.62	2.45 (0.24-24.61)	.45
Multiple systemic treatments	1.31 (0.10-16.50)	.83	0.49 (0.05-4.87)	.54
None	0.31 (0.05-2.03)	.22	1.53 (0.23-10.18)	.66
Previous radiotherapy	2.02 (0.45-9.01)	.36	8.58 (1.33-55.52)	.02
Dose fractionation^e^				
Moderate hypofractionation	1.93 (0.34-10.86)	.45	0.21 (0.05-0.83)	.03
Ultrahypofractionation	0.30 (0.06-1.47)	.14	0.22 (0.04-1.21)	.08
Planning target volume	1.00 (1.00-1.00)	.36	1.00 (1.00-1.00)	.42
Year since radiotherapy	0.73 (0.50-1.07)	.11	1.26 (0.86-1.83)	.24

Abbreviations: CVD, collagen vascular disorder; OR, odds ratio.

^a^ Logistic model was unstable because of overparameterization.

^b^ Relative to other CVD diagnoses.

^c^ Relative to breast site.

^d^ Relative to biologic treatment.

^e^ Relative to conventional fractionation.

## Discussion

In this cohort study of 197 patients with cancer and comorbid CVD, the unadjusted incidence of severe acute toxic effects was less than 10% across all dose fractionation groups. The incidences of severe late toxic effects were also low and were not substantially different among patients who received conventionally fractionated vs hypofractionated radiotherapy. To our knowledge, before the present analysis, few studies have characterized the association of dose fractionation with radiotherapy-associated toxic effects in patients with CVD. Morris and Powell^16^ reported no substantial consequences associated with dose per fraction in univariate analysis among a cohort of 209 patients with CVD who primarily received CF or MH radiotherapy regimens. A retrospective study by Lowell et al^29^ comprising 14 patients with CVD (8 patients with rheumatoid arthritis, 5 patients with systemic lupus erythematosus, and 1 patient with scleroderma) observed no grade 3, grade 4, or rare toxic effects associated with gamma knife radiosurgery for intracranial tumors after a median of 16 months of follow-up. The median size of treated tumors was 5.0 cm^3^ (range, 0.14-7.8 cm^3^), and the dose to 50% isodose threshold ranged from 12 Gy to 25 Gy in a single treatment fraction.

Dragun et al^30^ reported similar toxic effects and cosmetic profiles associated with adjuvant high-dose brachytherapy compared with other accelerated partial breast radiotherapy series among 9 women with early-stage breast cancer and CVD. All women were treated with multicatheter or balloon brachytherapy to 34 Gy in 10 fractions, for a median treatment volume of 45.5 cm^3^. Five of 9 women experienced grade 1 acute skin erythema, and 1 woman experienced wound dehiscence. Five women exhibited late skin induration, and 2 women experienced pain and telangiectasias. The CONTRAD meta-analysis^21^ included a modern series of contemporary radiotherapy techniques using both conventional and hypofractionated regimens. Although this meta-analysis did not specifically associate toxic effects with dose fractionation, it reported grade 3 or higher acute and late toxic effect rates of 11.7% (95% CI, 5.4%-19.6%) and 6.1% (95% CI, 1.4%-12.6%), respectively. Although no firm conclusions can be drawn from these studies, available data have thus far indicated low severe toxic effect rates across various dose fractionation regimens and are consistent with the findings from our study.

Despite the radiobiological principle that delivering a higher radiotherapy dose per fraction will increase the risk of damage to late-responding tissues, the incidences of severe toxic effects were low among patients receiving hypofractionated and conventionally fractionated regimens in our study. In the multivariable analysis, the receipt of MH radiotherapy was significantly associated with a lower likelihood of developing any late toxic effects, and the receipt of UH radiotherapy also indicated a lower likelihood. Technological advances in image guidance and target localization have enabled radiation oncologists to deliver hypofractionated regimens safely and with smaller margins, and the smaller treatment volumes could mitigate the potential risks of developing severe toxic effects. Despite the higher total biological effective dose delivered to the targeted site with hypofractionation, the better preservation of healthy tissues may mitigate the development of severe toxic effects; thus, no differences in severe acute or late toxic effects were observed in this study.

Broader use of hypofractionated regimens became possible after recent advancements in treatment techniques and image guidance. Therefore, time-dependent patterns of radiotherapy techniques and relatively shorter follow-up times with hypofractionation vs conventional fractionation could be confounding factors. In our study, patients receiving UH radiotherapy had a numerically (although not substantially) shorter follow-up time (16 months) compared with patients in the CF and MH groups (24 months). Treatment techniques were also not significantly associated with toxic effects or CVD symptom flares among patients in this study.

Efforts to characterize the toxic effects associated with radiotherapy by CVD subtype have been inconsistent in previous studies because of the heterogeneity and relative paucity of patients with cancer and comorbid CVD. Morris et al^16^ reported a greater risk of late toxic effects over a 5-year period among patients with CVD who did not have rheumatoid arthritis (91%) compared with those who had rheumatoid arthritis (56%; *P* = .002). In contrast, a matched-control study reported that no differences in acute or late toxic effects were observed between patients with and without CVD, although an increase in late toxic effects among patients with rheumatoid arthritis was noted.^18^ Patients with systemic lupus erythematosus experienced a lower incidence of late toxic effects but a greater number of acute toxic effects than those in the matched-control group.

Another matched-control study of 38 patients with CVD who did not have rheumatoid arthritis observed no difference in the incidence of acute or late toxic effects compared with patients in the control group without CVD; however, the researchers noted that patients with scleroderma had a greater incidence of grade 3 late toxic effects.^22^ Our study found that the incidence of severe acute toxic effects was similar across dose fractionation groups and CVD subtypes. Patients with psoriasis were observed to have an increased incidence of severe late toxic effects after receiving UH radiotherapy, while patients with rheumatoid arthritis experienced increases in severe late toxic effects after receiving CF radiotherapy.

Patients and physicians alike have raised concerns regarding radiotherapy exposure and exacerbation of CVD symptoms, yet few case studies were available to elucidate this issue. Localized radiotherapy is hypothesized to increase the expression and circulation of self-antigens via basement membrane damage, cellular apoptotic debris, or necrosis and is thought to exacerbate CVD symptoms overall. One case report by Robertson et al^31^ described 2 patients with rheumatoid arthritis and scleroderma who experienced progression of underlying CVD symptoms while also developing worse cosmetic outcomes after receiving breast radiotherapy. The patient with rheumatoid arthritis had a rheumatoid factor titer of 1:320 but negative antinuclear antibodies before radiotherapy. Her CVD symptoms worsened while developing radiotherapy-associated breast fibrosis, which required additional nonsteroidal anti-inflammatory medications. Before receiving radiotherapy, the patient with scleroderma experienced arthritis and skin thickening in her upper and lower extremities. After 27 months since the completion of radiotherapy, her scleroderma progressed to her esophagus.

In our study of 197 patients, CVD symptom flares occurred in approximately one-quarter of patients in each dose fractionation group, of which 52.3% adjusted their CVD medications to manage their symptoms. The proportion of CVD symptom flares in this cohort may be associated with CVD activity that occurred before the initiation of radiotherapy, as symptom flares were controlled or low among most patients in each fractionation group. In addition, these findings may be confounded by the progression of the CVD itself, independent of any cancer treatment.

### Limitations

This study has several limitations. The relative scarcity of patients with cancer and comorbid CVD limits the study of radiotherapy-associated toxic effects to an examination of retrospective reviews, which are dependent on the accuracy of physician reports. These assessments may be heterogeneous and biased. Recent studies have indicated that physicians often underestimate the adverse effects experienced by patients; therefore, the true incidence of toxic effects or CVD symptom flares could be higher across some or all dose fractionation groups.^32,33^ The comparison of toxic effects by dose fractionation group was not performed against a matched control group of patients without CVD. Although patients with cancer and CVD may potentially have a higher risk of experiencing late toxic effects compared with those without CVD, the unadjusted incidences of both acute and late adverse toxic effects across all dose fractionation groups in this study were similar to those found in previous matched-control studies and the general population.^18,22^ Moreover, the decision to prescribe a particular dose regimen is based on clinical indication and various patient and disease-specific factors.

This analysis included a heterogeneous number of CVD diagnoses, primary cancers, and treatment target sites. Each CVD diagnosis has a distinct natural history, relative radiosensitivity, and an extent and duration over which radiotherapy-associated toxic effects may develop and persist. Disease-modifying antirheumatic drugs are also variably used depending on CVD symptoms, with some drugs having relatively more radiosensitizing consequences than others.^34,35^ Hypofractionated radiotherapy is increasingly used for the treatment of certain cancers, such as prostate cancer, early-stage lung cancer, and select oligometastatic conditions, more than others. However, we were unable to evaluate the toxic effect incidence for every CVD diagnosis, primary cancer diagnosis, and treatment site because of the small subgroup samples and the likely difficulty in finding toxic effect differences with meaningful statistical power. Therefore, an evaluation of the safety of alternative dose fractionation regimens that includes these factors is still needed.

Toxic effect outcomes from this study are reflective of current radiotherapy regimens that are the standard of care for patients with specific cancer diagnoses and disease sites. Ongoing efforts to evaluate the safety of hypofractionation for nonstandard indications are presently being conducted for use in the general population and will need to continue to be used with caution, especially among patients with comorbid CVD. Overall, the use of alternative fractionation regimens requires individualization to each patient’s clinical situation.

## Conclusions

To our knowledge, this study is the first to examine the toxic effects associated with various dose-fractionated radiotherapy regimens for the treatment of patients with cancer and comorbid CVD in the context of modern technologies. The overall incidence of severe acute and late toxic effects across all dose fractionation groups was less than 10%. With recent technological advances enabling the delivery of high doses of radiation precisely and conformally, patients with CVD may not require immediate exclusion from the receipt of radiotherapy regimens as part of their cancer treatment when clinically indicated.

## Notes

Supplement. **eMethods.** Total Dose Comparison Using Biological Effective Dose
