# Supplementary material for: Assessment of Toxic Effects Associated With Dose-Fractionated Radiotherapy Among Patients With Cancer and Comorbid Collagen Vascular Disease
Source: JAMA Netw Open. 2021 Feb 18;4(2):e2034074. doi: 10.1001/jamanetworkopen.2020.34074 (PMC7893499; doi:10.1001/jamanetworkopen.2020.34074)
Supplement: Supplement. — eMethods. Total Dose Comparison Using Biological Effective Dose [file jamanetwopen-e2034074-s001.pdf]

Supplementary Online Content

Yoon SM, Chu F-I, Ruan D, Steinberg ML, Raldow A, Lee P. Assessment of toxic effects associated with dose-fractionated radiotherapy among patients with cancer and comorbid collagen vascular disease. *JAMA Netw Open*. 2021;4(1):e2034074. doi:10.1001/jamanetworkopen.2020.34074

eMethods. Total Dose Comparison Using Biological Effective Dose

This supplementary material has been provided by the authors to give readers additional information about their work.

© 2021 Yoon SM et al. *JAMA Network Open*.

eMethods. Total Dose Comparison Using Biological Effective Dose

Total radiation dose delivered to target and surrounding normal structures during radiotherapy was thought to be an important factor to consider for toxicity development. A common numeric score, the biologic effective dose (BED), was calculated to compare total dose delivered across various dose fractionation schemas. The equation for BED is outlined below:

$$BED = nd[1 + \frac{d}{\alpha/\beta}]$$

BED is biologic effective dose. *n* is number of radiation treatment fractions. *d* is dose per fraction. α/β is the dose where linear and quadratic cell killings are equal.

Early responding tissues to radiation have higher α/β ratios while late responding tissues have lower values. For our calculations, α/β ratio of 10 was used.

© 2021 Yoon SM et al. *JAMA Network Open*.
